# Influence of insulators on transgene expression from integrating and non-integrating lentiviral vectors

**DOI:** 10.1186/1479-0556-9-1

**Published:** 2011-01-04

**Authors:** Nicolas Grandchamp, Dorothée Henriot, Stéphanie Philippe, Lahouari Amar, Suzanna Ursulet, Che Serguera, Jacques Mallet, Chamsy Sarkis

**Affiliations:** 1 CRICM - Centre de Recherche de l'Institut du Cerveau et de la Moelle Epinière - UPMC/INERM UMR_S975/CNRS UMR7225, Equipe de Biotechnologie et Biothérapie, 83 boulevard de l'Hôpital, 75013 Paris, France; 2 NewVectys - 109 rue du Faubourg Saint-Honoré, 75008 Paris, France; 3 Unit of Gene Therapy & Stem Cell Biology, Ophthalmology Department of the University of Lausanne, Jules-Gonin Eye Hospital, avenue de France 15, 1004 Lausanne, Switzerland; 4 Neuronal Survival Unit, Department of Experimental Medical Science, Wallenberg Neuroscience Center, BMC A10, 221 84 Lund, Sweden; 5 CRC MIRcen - Laboratoire INSERM - Modélisation des biothérapies, 18, route du Panorama, 92265, Fontenay-aux-roses, France

## Abstract

**Background:**

The efficacy and biosafety of lentiviral gene transfer is influenced by the design of the vector. To this end, properties of lentiviral vectors can be modified by using *cis*-acting elements such as the modification of the U3 region of the LTR, the incorporation of the central *flap *(cPPT-CTS) element, or post-transcriptional regulatory elements such as the woodchuck post-transcriptional regulatory element (WPRE). Recently, several studies evaluated the influence of the incorporation of insulators into the integrating lentiviral vector genome on transgene expression level and position effects.

**Methods:**

In the present study, the influence of the matrix attachment region (MAR) of the mouse *immunoglobulin-κ (*Ig-κ) or the chicken *lysozyme *(ChL) gene was studied on three types of HIV-1-derived lentiviral vectors: self-inactivating (SIN) lentiviral vectors (LV), double-copy lentiviral vectors (DC) and non-integrating lentiviral vectors (NILVs) in different cell types: HeLa, HEK293T, NIH-3T3, Raji, and T Jurkat cell lines and primary neural progenitors.

**Results and Discussion:**

Our results demonstrate that the Ig-κ MAR in the context of LV slightly increases transduction efficiency only in Hela, NIH-3T3 and Jurkat cells. In the context of double-copy lentiviral vectors, the Ig-κ MAR has no effect or even negatively influences transduction efficiency. In the same way, in the context of non-integrating lentiviral vectors, the Ig-κ MAR has no effect or even negatively influences transduction efficiency, except in differentiated primary neural progenitor cells.

The ChL MAR in the context of integrating and non-integrating lentiviral vectors shows no effect or a decrease of transgene expression in all tested conditions.

**Conclusions:**

This study demonstrates that MAR sequences not necessarily increase transgene expression and that the effect of these sequences is probably context dependent and/or vector dependent. Thus, this study highlights the importance to consider a MAR sequence in a given context. Moreover, other recent reports pointed out the potential effects of random integration of insulators on the expression level of endogenous genes. Taken together, these results show that the use of an insulator in a vector for gene therapy must be well assessed in the particular therapeutic context that it will be used for, and must be balanced with its potential genotoxic effects.

## Background

Lentiviral vectors are among the best gene transfer tools for both dividing and non-dividing cells. Their relatively recent development has been underpinned by accumulated understanding of the biology of the human immunodeficiency virus (HIV) and experience with oncoretrovirus-derived vectors. The biosafety of gene transfer tools depends in part on their efficacy, and efficacy can be optimized by rational vector design. Over the past ten years, many improvements have been made to lentiviral vector systems so as to improve their biosafety and performance.

The effects of various *cis*-acting modifications have been evaluated as a means to increase the transduction efficiency of lentiviral vectors and consequently reduce the amount of vector needed for efficient transduction. Self-inactivating (SIN) vectors with deletions in the U3 enhancer region of the LTR (Long Terminal Repeat) have been developed and display higher biosafety, through abolition of the enhancer activity, and stronger transgene expression than the unmodified parental vectors both in MLV- and HIV-1-derived vectors [1-5]. The incorporation of the lentiviral *flap *sequence, or *cPPT-CTS*, enhances transduction efficiency by 2 to 10 fold in many cell types both *in vitro *and *in vivo*, proportionally reducing the quantity of vector needed for high frequency transduction [6-9]. The incorporation of the regulatory sequence WPRE [10,11] or the 3' UTR of the *tau *or t*yrosine hydroxylase *genes into the transgene expression cassette also enhances transgene expression by several fold [12]. S/MAR (Scaffold/Matrix Attachment Region) and LCR (Locus Control Region) are insulators, and their contribution to expression has been studied in the context of LV. Insulators are DNA sequence elements that prevent inappropriate interactions between adjacent chromatin domains (for review see [13]). The Ig-κ gene MAR, but not the chicken lysozyme gene MAR, has been reported to enhance transgene expression in hepatic cells by about 4-fold both *in vitro *and *in vivo *[14]. The incorporation of a SAR from the human interferon-β gene into SIN lentiviral vector backbone increases average GFP expression in human ES cells [15] and human CD34+ hematopoietic cells [16]. The inclusion of the SAR together with the LCR (5'HS4) from the chicken β-globin locus reduced the variability in GFP expression, *i.e*. repressive position effects, in human ES cells [15] and human CD34+ hematopoietic cells [16]. The LCR (5'HS4) from the chicken β-globin locus has also been reported to prevent, partially or fully, positional effects on retrovirus-driven transgene expression in erythropoietic cells [17,18]. However, this could not be confirmed in another context, where the same sequences had no effect in dividing RN33B neural stem cells [19].

Another factor that may influence the use of insulators for gene transfer is their position in the vector backbone, and more specifically their presence on both sides of the expression cassette. Indeed, MARs have been shown to be more effective when flanking the transgene expression cassette by preventing positional effects and by preventing negative epigenetic modifications of the integrated DNA [20]. In oncoretroviral and LV, a simple way to obtain vectors where a MAR flanks the expression cassette is to clone it in place of the U3 region of the 3'LTR. After reverse transcription, the MAR is copied into the U3 region of the 5'LTR giving rise to a proviral genome that contains the expression cassette flanked by the MAR. Recent studies demonstrated that the insertion of the 1.2 kb HS4 MAR sequence in the U3 region of a DC lentiviral vector can reduce the RT process and consequently reduce the titer and efficacy of the vector [21-23]. However, this effect was not observed with a 250 bp MAR sequence [21].

Because insulators can affect the expression of genes placed at long distance, it is also important to carefully consider the potential genotoxic effect of MARs when placed in a vector leading to integration of the MAR into the target cell genome. This is all the more important as integration of lentiviral vectors is preferentially targeted in active transcription units, making the lentivirally-driven integration of MARs potentially genotoxic. For instance, in the Burkitt's lymphoma, expression of c-myc gene is deregulated by its translocation near the HS4 region of the murine immunoglobulin heavy chain. In an *in vitro *study, using a luciferase reporter system, it has been shown that the murine HS4 region activates the *c-myc *promoter activity by 46-fold and the human HS4 region by 14-fold [24]. Moreover, a recent work showed that aberrant expression of the gene that encodes the STAB1 protein, which binds to insulator sequence, was responsible for the generation of brain tumors [25]. However, in the context of integrating lentiviral gene transfer, the genotoxicity issue has been studied relatively little, and few recent reports gave rise to contradictory conclusions [26-29].

A solution to the potential genotoxicity of LV was the development of non-integrative lentiviral vectors (NILVs), as it was shown by us [30] and other groups [31,32]. These vectors remain as episomal genomes in the nucleus of the transduced cells (for review see [33-35]) and therefore avoid the risk of genotoxicity by insertional mutagenesis. They have great potential for clinical use, particularly in non-dividing cells where their episomal genome remains stable for at least one year [32]. However, transgene expression from such vectors may be 2 to 10 times less strong than that from otherwise similar integrative control vectors [1,30,36,37]. It would therefore be valuable to improve the transduction efficiency of NILVs so as to reduce the quantity of vector needed. Although insulators have been studied in integrating lentiviral vectors, their effects on transgene expression from NILVs have never been investigated.

We incorporated the MARs from the immunoglobulin-κ (Ig-κ) and the chicken lysozyme genes into three lentiviral vector backbones (SIN, DC and NILV) and assessed the effects *in vitro *in several cell types. The presence of these MARs in SIN lentiviral vectors, DC lentiviral vectors and NILVs did not result in significant or relevant systematic enhancement of transgene expression in the cell types tested, and indeed, in some cases led to a decrease in transgene expression.

## Methods

### Plasmids

Vector design is summarized in the Additional File 1.

Encapsidation plasmids expressing a functional integrase (p8.91 IN_WT_) or a N mutant integrase (p8.91 IN_N_) have been described previously [30]. The N substitution consists of the replacement of the 262RRK motif of the N region of the integrase (IN) coding sequence with AAH, the equivalent motif of the Moloney murine leukemia virus IN.

The immunoglobulin gamma (Ig-κ) gene MAR sequence was amplified by PCR from genomic DNA of C57B6 mice (Genbank sequence V00777, nucleotides 3345 to 3758) with the following primers, which created NheI and SalI restriction sites at the 5' and 3' ends, respectively, of the Ig-κ MAR sequence: mar1: 5'GGCTAGCAGGGCATAAACTGCTTTATCCAGTG3'; mar2: 5'CGTCGACATAACTTAATGACTCTAAAGTAGTTTC3'. The PCR product was introduced in place of the NheI-SalI fragment of the previously described pTrip-CMV-GFP-WPRE [30] to generate Trip- MAR_Igκ_-CMV-GFP-WPRE.

The plasmids pTrip-CMV-LUC-WPRE and Trip- MAR_Igκ_-CMV-LUC-WPRE were generated by replacing GFP sequence (XhoI-SpeI fragment) in pTrip-CMV-GFP-WPRE and Trip-MAR_Igκ_-CMV-GFP-WPRE, respectively, with the luciferase sequence (XhoI-XbaI fragment from pGL3 (Promega)).

The plasmid pTrip-EF1-LUC-MAR_IgK_dc-SIN is derived from pTrip-EF1-EGFP-SIN in which a multiple cloning site has been inserted in place of the U3 deletion in the 3' LTR. The EGFP sequence (BsrGI- XhoI fragment) was replaced with the luciferase sequence (BsrGI-BamHI fragment from plasmid pGL3 (Promega)) to generate pTrip-EF1-LUC-dc-SIN. The MAR Igκ sequence was inserted in place of the NheI-SalI fragment in pTrip-EF1-LUC-SIN to generate pTrip-EF1-LUC-MAR_IgK_dc-SIN.

The plasmids Trip-MAR_ChLS_-CMV-LUC-WPRE and Trip-MAR_ChLAS_-CMV-LUC-WPRE were constructed by introducing the MAR sequence from the chicken lysozyme (ChL) gene (SmaI-BsrBI fragment) from the previously described pPGA1 [38]) into the SalI restriction site in pTrip-CMV-LUC-WPRE in sense and anti-sense orientations, respectively.

### Lentiviral vector production and purification

Lentiviral vectors were generated by transient transfection of 293T cells by the calcium phosphate precipitation method as described previously [30]. For all experiments, the LUC vector with the MAR sequence and the corresponding control LUC vector without the MAR sequence were produced simultaneously. Vectors were titrated by assaying HIV p24 Gag antigen in each stock by ELISA (HIV-1 P24 antigen assay; Beckman Coulter, Fullerton, CA).

### Cell lines and primary cultures

Human epithelial HeLa and HEK293T cells and murine NIH-3T3 cells were grown in Dulbecco's modified medium supplemented with 100 U/mL penicillin, 100 μg/mL streptomycin and 10% fetal calf serum (FCS). The human Jurkat T-cell line and human Raji B-cell line were cultured in RPMI 1640 medium containing 10% FCS, 1% HEPES, 1% glutamine, 100 U/ml penicillin and 100 mg/ml streptomycin. Neural telencephalic progenitor cultures were generated and maintained as described previously [39].

### Transduction of cells

HEK293T, Hela and NIH-3T3 cells were seeded at densities of 15,000, 5,000 and 6,500 cells per well, respectively, in 96-well plates. The cells were transduced 24 hours later in medium supplemented with 1 μM DEAE-Dextran. Contact with the vectors was allowed for 4 hours then the medium was removed and replaced with fresh medium. Raji and Jurkat cells were seeded in 24-well plates at a density of 100,000 cells per well. These cells were infected and maintained in RPMI 1640 medium supplemented with 10% FCS, 1% HEPES, 1% glutamine and 100 μg/mL each penicillin and streptomycin. After 12 hours of contact with the virions, the cells were washed and the RPMI medium was replaced.

Neural progenitor cells were seeded in 96-well plates coated with an adherent substrate (gelatin and laminin) at a density of 10,000 cells per well in a N2 standard medium supplemented with 10 ng/ml bFGF (Roche Diagnostics, Nutley, NJ). These cultures were maintained for 2 days then transduced with various doses of vector. After 24 hours of incubation with the vector, the medium was replaced with either standard medium (N2 + bFGF) or standard medium supplemented with 10% fetal calf serum to induce glial differentiation.

### Luciferase assay

Luciferase activity was measured 72 h after transduction using the Promega Bright-Glo Luciferase Assay Kit according to the manufacturer's protocol. The cells were rinsed with 1X PBS and 100 μl of Glo Lysis Buffer was added directly in the wells. The plates were incubated for 5 minutes at room temperature, then the lysate transferred into 0.5 ml Eppendorf tubes and used directly for luciferase activity assay or stored at -80°C. The tubes were incubated for 5 minutes at room temperature and stored at -80°C. The firefly luciferase activity assay was performed following the manufacturer's instructions by adding 10 μl of Bright-Glo™Assay Reagent to an equal volume of sample and the luminescence was measured with a luminometer.

### Statistical analyses

**Figure 1:**
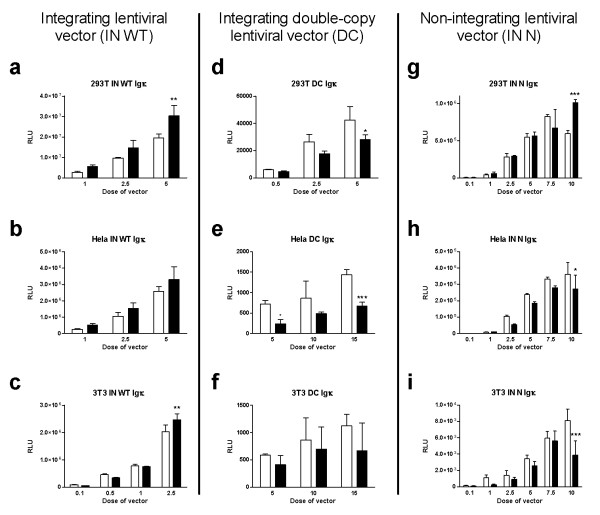
**Effects of the Igκ MAR in three lentiviral vectors**. Three types of cells (HEK293T, Hela and NIH-3T3) were transduced in triplicate with a series of doses of the vector (ranging from 0.1 to 15 ng of p24, measured by ELISA), and luciferase activity was measured 72 hours later. HEK293T, Hela and NIH-3T3 cells were seeded at densities of 15,000, 5,000 and 6,500 cells per well, respectively, in 96-well plates. SIN integrating (IN WT) lentiviral vectors bearing a CMV-LUC expression cassette, double-copy (DC) integrating lentiviral vectors bearing an EF1-LUC expression cassette and non-integrating (IN N) lentiviral vectors bearing a CMV-LUC expression cassette were used without (white columns) or with (black columns) the Igκ MAR. Statistical analysis was performed using a two-way ANOVA, and statistical significance of the Bonferroni post-test is represented on the relevant bars (* for p < 0.05, ** for p < 0.01 and *** for p < 0.001).

**Figure 2:**
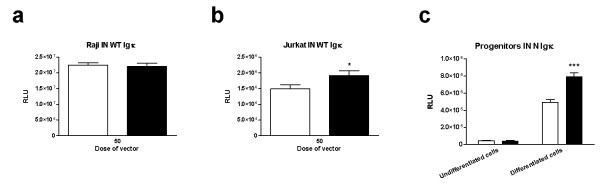
**Effects of the Igk MAR in lymphoblastoma and neural progenitor cells**. Raji (a), Jurkat (b) and neural progenitor (c) cells were transduced using SIN integrating (IN WT) or non-integrating (IN N) lentiviral vectors expressing the luciferase transgene with (black columns) or without (white columns) the Igκ MAR. Raji and Jurkat cells were seeded in 24-well plates at a density of 100,000 cells per well. Neural progenitor cells were seeded in 96-well plates at a density of 10,000 cells per well in a N2 standard medium supplemented with 10 ng/ml bFGF. After contact with the vectors, neural progenitors were maintained undifferentiated or were glially differentiated by addition of 10% fetal calf serum in the culture medium. Unpaired t test was performed to analyze results of figures a and b and two-way ANOVA for figure c. Statistical significance of the t test (figure b, * for p < 0.05) or the Bonferroni post-test (figure c, *** for p < 0.001) are represented on the relevant columns.

**Figure 3:**
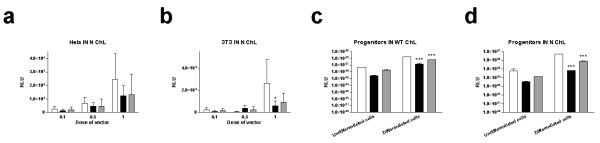
**Effects of the chicken lysozyme (ChL) gene MAR on integrating (IN WT) and non-integrating (IN N) lentiviral vectors**. Hela (a) and NIH-3T3 (b) and neural progenitor (c and d) cells were transduced with integrating (IN WT) or non-integrating (IN N) vectors containing a luciferase transgene expression cassette without (white columns) the ChL MAR or with a sense-oriented (black columns) or antisense-oriented (grey columns) ChL MAR. Statistical analysis was performed using a two-way ANOVA, and statistical significance of the Bonferroni post-test is represented on the relevant bars (* for p < 0.05, ** for p < 0.01 and *** for p < 0.001).

GraphPad Prism 5 software (GraphPad software, Inc) was used for all statistical analyses. Results were analyzed using two-way ANOVA followed by Bonferroni post-tests for results reported in Figures 1, 2 and 3, except for the results reported in Figures 2a and 2b for which an unpaired t test was used.

## Results and discussion

We studied the effects of the incorporation of the Igκ MAR on transgene expression from three types of LV containing a luciferase expression cassette (see Additional File 1 for vector design). Three types of cells (HEK293T, Hela and NIH-3T3) were transduced in triplicate with a series of doses of the vector, and luciferase activity was measured 72 hours later. In LV, luciferase activity was significantly enhanced by the presence of the Igκ MAR in HEK293T (Figure 1a, two-way ANOVA, p = 0.0004) and Hela cells (Figure 1b, two-way ANOVA, p = 0.0190), but not in 3T3 cells (Figure 1c, two-way ANOVA, p = 0.2214). The enhancement of expression by Igκ MAR was however moderate, and always less than double; these findings are discordant with those previously described for hepatic cells by Park and Kay [14], who reported about 4-fold increase of transgene expression level using the same Igκ MAR.

MARs have been shown to be more effective in some cases when flanking the transgene expression cassette [20]. We therefore constructed DC in which the Igκ MAR was inserted into the U3 region of the 3'LTR which results after RT in an integrative vector flanked by the MAR inserted in both U3 regions. Surprisingly, transgene expression from the DC was significantly weaker than that from control vectors without MAR, in both HEK293T cells (Figure 1d, two-way ANOVA, p = 0.0041) and Hela cells (Figure 1e, two-way ANOVA, p < 0.0001); there was no difference between MAR DC vectors and control vectors in NIH-3T3 cells (Figure 1f, two-way ANOVA, p = 0.1167). Thus, the presence of two copies of Igκ MAR flanking the transgene does not enhance expression in these cells, but decreases expression by up to about 50%. This may be because the MAR-containing DC vectors are longer than the control constructs; increased length may reduce the encapsidation efficiency [23,40,41] or result in lower processing by the HIV reverse transcriptase, which is a poorly efficient enzyme [42,43]. This was confirmed by recent studies demonstrating that the insertion of a 1.2 kb HS4 MAR in the U3 region of an integrative LV can reduce the RT process and consequently reduce the titer of the vector [21-23]. However, this effect was influenced by the length of the incorporated sequence as the negative effect could not be observed with a 250 bp sequence corresponding to the core element of the HS4 MAR [21]. Our results suggest the negative effect on RT processing could already occur with a 420 bp sequence.

To test the effects of the Igκ MAR in an episomal context, we produced NILVs containing the Igk MAR and used these constructs to transduce HEK293T, HeLa and NIH-3T3 cells. Unlike what we observed with integrative vectors, Igκ MAR significantly reduced luciferase expression in Hela (Figure 1h, two-way ANOVA, p = 0.0013) and NIH-3T3 (Figure 1i, two-way ANOVA, p = 0.0004) cells. In HEK293T cells, MAR did not significantly affect expression (Figure 1g, two-way ANOVA, p = 0.0647), except at the highest dose of vector (Figure 1g; at the highest dose about 60% stronger expression than the control vector; Bonferroni post-test p < 0.001). In conclusion, the Igk MAR does not generally enhance transgene expression from an episomal lentiviral vector.

The cell-type may determine the effects of the MAR, so we investigated its effects in cells in which immunoglobulin-κ chains are normally expressed, *i.e*. lymphocytes. We transduced Raji (human B lymphoblastoma cells) and Jurkat cells (human T lymphoblastoma cells) with an integrating LV expressing luciferase, with or without the Igκ MAR. In Raji cells, the MAR did not influence luciferase expression at all (Figure 2a, unpaired t test, p = 0,5998), whereas in Jurkat cells the presence of MAR was associated with a small but significant increase in expression (28%, unpaired t test, p = 0.0228; Figure 2b). Thus, the presence of Igκ MAR in an LV does not lead to a large increase of transgene expression in lymphocytic cells.

MARs facilitate transcription by epigenetic mechanisms involving chromatin remodelling, histone hyperacetylation and DNA demethylation [20]. We therefore tested the influence of the Igκ MAR in a cell culture in which transgene expression from lentiviral vectors is strongly repressed by epigenetic inhibition. Expression from a lentiviral vector in undifferentiated neural progenitor cells is greatly enhanced after serum-induced differentiation of these cells into the glial fate [39]. We used this model, and first confirmed that epigenetic repression inhibited transgene expression from lentiviral vectors: we transduced neural progenitor cell cultures with LV expressing GFP (data not shown) or luciferase (see Additional File 2) and treated the cells with sodium butyrate, an inhibitor of histone deacetylases (treatment with sodium butyrate leads to a massive histone hyperacetylation and generally induces expression from silenced genes). Following treatment with sodium butyrate, luciferase expression increased substantially (over 10-fold increase in undifferentiated cells), confirming the strong epigenetic repression of transgene expression from our lentiviral vector in these cells (see Additional File 2). We then transduced undifferentiated and serum-differentiated neural progenitor cultures with NILVs (with and without the Igκ MAR) and assayed transgene expression. Transgene expression was significantly (about 60%) higher from vectors with than without the MAR (Figure 2c, two-way ANOVA, p < 0.0001) only in glially differentiated cultures (Figure 2c, Bonferroni post-test, p < 0.001) and not in undifferentiated cultures (Figure 2c, Bonferroni post-test, p > 0.05). Thus the observed moderate MAR-associated increase in expression was independent of epigenetic repression, and appeared to be a cell-type specific effect.

We tested the effects of another insulator, the chicken lysozyme (ChL) gene MAR. Luciferase-expressing LV and NILV lentivectors were produced, containing or not the ChL MAR, incorporated in sense or antisense orientation upstream of the cPPT-CTS region (see Additional Figure 1). In Hela cells, the presence of the ChL MAR in a NILV did not significantly affect the transgene expression (Figure 3a, two-way ANOVA, p = 0.4199). The same result was observed in NIH-3T3 cells (Figure 3b, two-way ANOVA, p = 0.2349), and the sense-oriented MAR even led to a ~70% decrease of the transgene expression at the highest dose (Figure 3b, Bonferroni post-test, p > 0.05). In progenitor cell cultures, the ChL MAR had large and significant negative effects on transgene expression from both integrating and NILVs. In the integrating vector, the significance was very high (Figure 3c, two-way ANOVA, p < 0.0001) especially in differentiated cells in which the decrease of transgene expression was up to ~11-fold (Figure 3c, sense ChL, Bonferroni post-test, p < 0.001 and antisense ChL, Bonferroni post-test, p < 0.001). In the NILV, ChL MAR similarly reduced expression with high statistical significance (Figure 3d, two-way ANOVA, p < 0.0001) especially in glially differentiated cells (Figure 3d, sense ChL, Bonferroni post-test, p < 0.001 and antisense ChL Bonferroni post-test, p < 0,001).

## Conclusion

In conclusion, the Igκ MAR does not systematically increase transgene expression from lentiviral vectors -- whether integrative, double-copy or non-integrating -- in the cell lines tested, or even in lymphocytic cells or epigenetically repressed cells. The ChL MAR may either not affect transgene expression or have moderate or strong negative effects on transgene expression, depending on the cell type. These results are summarized in the following Table 1:

**Table 1:** Summary of statistically relevant effects of Igκ and ChL MARs on transduction efficiency on various cells in integrating (LV), double-copy (DC) and non-integrating (NILV) lentiviral vectors. (+) positive effect, (-) negative effect, (0) no effect.

		Vectors				
	
		LV Igκ	DC Igκ	NILV Igκ	LV ChL	NILV ChL
Cells	293T	+	-	+		
	
	Hela	0	-	-		0
	
	3T3	+	0	-		-
	
	Primary neural progenitors undifferentiated			0	0	0
	
	Primary neural progenitors differentiated			+	-	-
	
	Raji	0				
	
	Jurkat	+				

Our findings highlight the importance of studying the effects of particular MARs in appropriate model systems as they may not lead to the expected increase of transgene expression. It seems that alternative ways to enhance transgene expression are required, for example using strong promoters, *cis*-acting non-coding sequences [12] or, as was very recently demonstrated for NILVs in some cell types, by optimizing the vector backbone by deleting particular parts of the U3 region [1].

## List of abbreviations

ANOVA: ANalysis Of VAriance; ChL: Chiken Lysozyme; cPPT-CTS: Central Polypurine Tract-Central Termination Sequence; DC: Double Copy Lentiviral Vector; ELISA: Enzyme Linked ImmunoSorbent Assay; GFP: Green Florescence Protein; HIV: Human Immunodeficiency Virus; HS4: Hypersensitive Site 4; Ig-κ: immunoglobulin-κ; IN: Integrase; LTR: Long Terminal Repeat; LUC: Luciferase; LV: Integrating Lentiviral Vector; MAR: Matrix Attachment Region; NILV: Non Integrative Lentiviral Vector; PCR: Polymerase Chain Reaction; RLU: Relative Light Unit; RT: Reverse Transcriptase; SAR: Scaffold Attachment region; SIN: Self Inactivating; STAB1: Special AT-rich Sequence Binding protein 1; UTR: Untranslated Region

## Competing interests

N.G. D.H, S.U. and C.Sa. are members of NewVectys, which owns the commercialization rights of the NILVs. S.P, J.M, C.Se. and C.Sa are listed as inventors on patent applications related to NILVs. These conditions do not alter the authors' adherence to Genetic Vaccines and Therapy policies. Materials and information associated with the authors' publication will be freely available to those as reasonably requested for the purpose of academic, non-commercial research.

## Authors' contributions

Conceived, designed and performed the experiments: NG, DH, SP, LA., SU, CSe and CSa. Supervised the work : CSa and JM. Participated to the article writing: CSa, NG and JM. All authors read and approved the final manuscript.

## Authors' information

Current address of S.P.: Unit of gene therapy and stem cell biology Jules-Gonin Eye Hospital, 15 avenue de France 1004 Lausanne, Switzerland.

Current address of L.A.: Neuronal Survival Unit, Department of Experimental Medical Science, Wallenberg Neuroscience Center, Lund University, 221 84 Lund, Sweden.

Current address of C.Se.: MIRCen laboratoire INSERM - Modélisation des Biothérapies -18 route du Panorama, Fontenay-aux-Roses, 92265 France.

All the authors read and approved the final manuscript.

## Supplementary Material

Additional File 1 **Plasmids used for lentiviral production**. Three plasmids are cotransfected in HEK293T cells for vector production. The vector plasmid contains the expression cassette and a MAR subcloned upstream the flap (cPPT-CTS) sequence, in sense (Igk or ChL MAR) or antisense (ChL) orientation. For double-copy vectors, the Igk MAR is subcloned in place of the U3 region in the 5' LTR, in sense orientation. The encapsidation plasmid contains the gag and pol genes. For the production of the non-integrative lentiviral vectors, the pol gene is mutated within the integrase coding sequence (^262^AAH substitution). For the production of integrative (SIN) or double-copy (DC) vectors, the WT integrase sequence is used. The envelope plasmid contains an expression cassette of the VSV envelope glycoprotein under the control of a CMV promoter. Click here for file

Additional File 2 **Effect of differentiation of neural progenitor cells on lentiviral transduction efficiency**. Neural progenitor cells were transduced with a luciferase expressing lentiviral vector (integrating) and kept in medium keeping them in an undifferentiated state or glially differentiated state (by addition of 10% FCS). Differentiation of the cells by FCS leads to an increase of the transgene expression. Moreover, the addition of butyrate (5 mM) in the medium after transduction leads to a high enhancement of expression, particularly in undifferentiated cells, highlighting strong negative epigenetic regulation of the transgene. Click here for file
