# Supplementary material for: Influence of insulators on transgene expression from integrating and non-integrating lentiviral vectors
Source: Genet Vaccines Ther. 2011 Jan 4;9:1. doi: 10.1186/1479-0556-9-1 (PMC3025823; doi:10.1186/1479-0556-9-1)

Lentivirus Vector plasmids: pTrip-Igκ, pTrip-ChL$_S$ and pTrip-ChL$_{AS}$

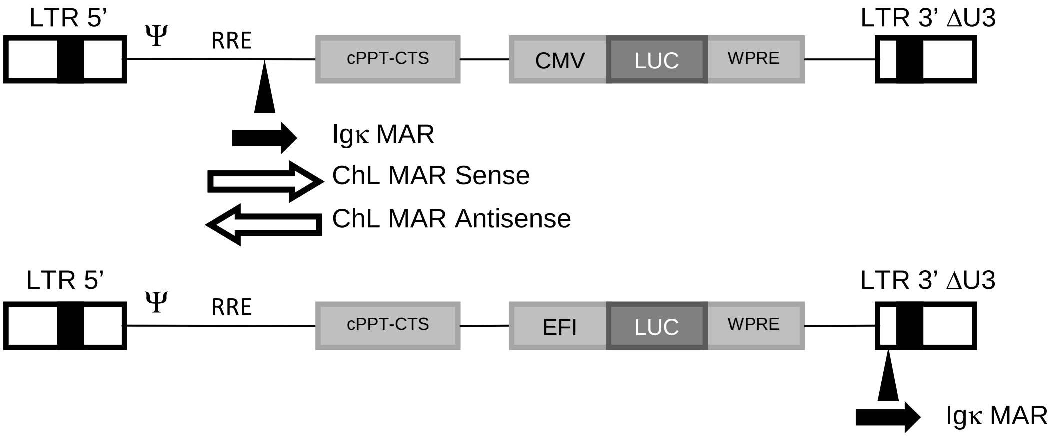

Encapsidation plasmids: p8.9 IN WT and p8.9 IN N

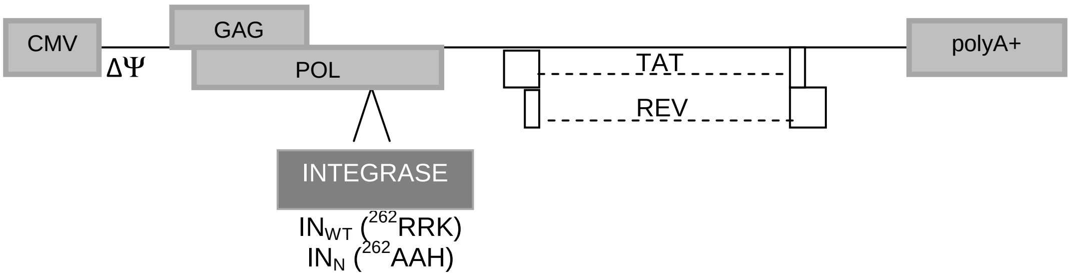

Envelope plasmid: pVSV-G

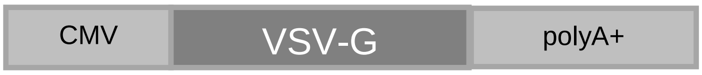

Supplement: Additional File 1 — Plasmids used for lentiviral production. Three plasmids are cotransfected in HEK293T cells for vector production. The vector plasmid contains the expression cassette and a MAR subcloned upstream the flap (cPPT-CTS) sequence, in sense (Igk or ChL MAR) or antisense (ChL) orientation. For double-copy vectors, the Igk MAR is subcloned in place of the U3 region in the 5' LTR, in sense orientation. The encapsidation plasmid contains the gag and pol genes. For the production of the non-integrative lentiviral vectors, the pol gene is mutated within the integrase coding sequence (262AAH substitution). For the production of integrative (SIN) or double-copy (DC) vectors, the WT integrase sequence is used. The envelope plasmid contains an expression cassette of the VSV envelope glycoprotein under the control of a CMV promoter. [file 1479-0556-9-1-S1.PDF]
