# Supplementary figures and images for: Influence of insulators on transgene expression from integrating and non-integrating lentiviral vectors
Source: Genet Vaccines Ther. 2011 Jan 4;9:1. doi: 10.1186/1479-0556-9-1 (PMC3025823; doi:10.1186/1479-0556-9-1)

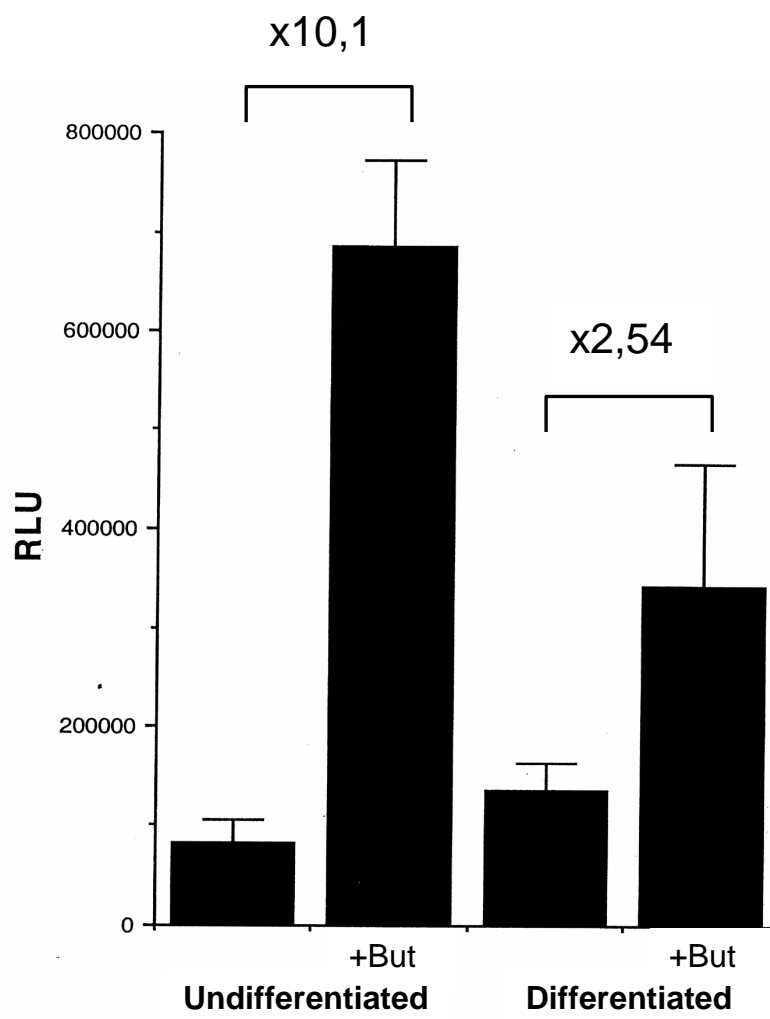

Supplement: Additional File 2 — Effect of differentiation of neural progenitor cells on lentiviral transduction efficiency. Neural progenitor cells were transduced with a luciferase expressing lentiviral vector (integrating) and kept in medium keeping them in an undifferentiated state or glially differentiated state (by addition of 10% FCS). Differentiation of the cells by FCS leads to an increase of the transgene expression. Moreover, the addition of butyrate (5 mM) in the medium after transduction leads to a high enhancement of expression, particularly in undifferentiated cells, highlighting strong negative epigenetic regulation of the transgene. [file 1479-0556-9-1-S2.PDF]
